# Full-Genome Sequence of Infectious Laryngotracheitis Virus (Gallid Alphaherpesvirus 1) Strain VFAR-043, Isolated in Peru

**DOI:** 10.1128/genomeA.00078-18

**Published:** 2018-03-08

**Authors:** Sandra Morales Ruiz, Jorge Bendezu Eguis, Ricardo Montesinos, Luis Tataje-Lavanda, Manolo Fernández-Díaz

**Affiliations:** a Laboratorios de Investigación y Desarrollo, FARVET SAC, Chincha Alta, Ica, Peru; b FARVET SPF SAC, Chincha Alta, Ica, Peru

## Abstract

We report here the first genome sequence of infectious laryngotracheitis virus isolated in Peru from tracheal tissues of layer chickens. The genome showed 99.98% identity to the J2 strain genome sequence. Single nucleotide polymorphisms were detected in five gene-coding sequences related to vaccine development, virus attachment, and viral immune evasion.

## GENOME ANNOUNCEMENT

Infectious laryngotracheitis is an avian respiratory disease which is caused by infectious laryngotracheitis virus (ILTV), and clinical signs vary from coughing, conjunctivitis, and gasping to expectoration of bloody mucus and death (1).

Whereas genomic database analysis allowed the identification of an ILTV field strain in Australia as a causative agent of outbreaks as a result of recombination between attenuated vaccine viruses (2), genomic sequence data from viral outbreaks in South America are still unavailable.

ILTV was reported for the first time in Peru by the Servicio Nacional de Sanidad Animal (SENASA) in 2008 (3). ILTV in Peruvian poultry has been associated with a 16% decrease in egg production, an 18% increase in layer chicken mortality (3), and a 27.3% decrease in productive efficiency in broilers (4).

In December 2014, an ILT outbreak occurred at a farm in Chincha Alta, Ica, Peru. An ILTV field strain named VFAR-043 was isolated from tracheal tissues and propagated onto the chorioallantoic membrane (CAM) of specific-pathogen-free eggs (5). This animal study was approved by the institutional ethics committee of the Universidad Peruana Cayetano Heredia, Lima, Peru (SIDISI 65330). Total genomic DNA was extracted from the suspension of the inoculated CAMs using a phenol-chloroform method (6).

Whole-genome sequencing was performed by Macrogen, Inc. (South Korea), using a HiSeq 2000 platform. The reads were assembled *de novo* using NextGene (7), with a coverage of 87×. The open reading frames (ORFs) were analyzed and annotated using SnapGene (GSL Biotech LLC) and Artemis (8). BLAST (9) was performed to determine gene identity. The genome of VFAR-043 was 152,701 bp long. The genome was composed of a unique long (UL) sequence and a unique short (US) sequence, which were 113,917 bp and 13,125 bp, respectively. The US sequence was flanked by inverted internal repeat (IR) and terminal repeat (TR) sequences of 12,829 bp and 12,830 bp, respectively. The G+C content was 48.06%, and the genome encodes 79 predicted ORFs.

VFAR-043 showed 99.98% identity to the J2 strain, which was reported as a possible parental virus of American strains (10). A 98.85% identity was obtained as a result of alignment against the genome sequence of strain 4787/80 isolated from Italy, which has been associated with an attenuated vaccine revertant introduced into Europe from America (11). Moreover, VFAR-043 shared 99.47% identity with the Australian reference vaccine SA2 strain.

We found nonsynonymous single nucleotide polymorphisms (nsSNPs) in 5 gene-coding sequences (CDS) of VFAR-043 in comparison to the SA2 strain. We detected 4 nsSNPs in glycoprotein B (gB) (UL27), a protein which is associated with viral attachment to cells (11); 2 nsSNPs for gE (US8), a protein which has a role in the cell-to-cell spread of ILTV (12); 6 nsSNPs for gD (US6), a protein which is a receptor for ILTV binding and entry to susceptible cells (11); 8 nsSNPs in gG (US4), which is a chemokine-binding protein (13); and 10 nsSNPs for gJ (US5), a protein which blocks the apoptotic cascade in host cells (11).

To our knowledge, this work presents the first ILTV genome sequence reported in South America; therefore, this study will allow us to clarify ILTV epidemiology in Peru and to contribute to the knowledge of this virus in this region.

### Accession number(s).

This full-genome sequence of the VFAR-043 strain has been deposited to GenBank under accession no. MG775218. The version described in this paper is the first version, MG775218.1.
